# Cemento-Osseous Dysplasia: A Detailed Comparison of the 2005 and 2017 WHO Classifications and Case Analysis

**DOI:** 10.7759/cureus.49041

**Published:** 2023-11-19

**Authors:** Jiankang Zhang, Yunbo Yu, Wei Tang, Jian Pan, Wei Jing

**Affiliations:** 1 Department of Oral and Maxillofacial Surgery, State Key Laboratory of Oral Diseases & National Clinical Research Center for Oral Diseases, West China Hospital of Stomatology, Sichuan University, Chengdu, CHN; 2 Department of Oral and Maxillofacial Surgery, Stomatological Hospital of Chongqing Medical University, Chongqing, CHN

**Keywords:** oral surgery, sclerotic bone lesions, alveolar bone resorption, who classification, cemento-osseous dysplasia

## Abstract

The WHO substantially redefined cemento-osseous dysplasia (COD) in 2017. The descriptions of COD in the 2005 and 2017 WHO classifications are quite different. In this study, we compared the difference in COD description between the 2005 and 2017 editions of the WHO classifications in detail. There are remarkable differences in the terminology, definition, synonyms, epidemiology, classifications, clinical features, radiation/pathology, prognosis, and predictive factors of COD between the two versions. At present, the surgical treatment of COD is less defined, and there is no clear guidance for the treatment of sclerotic bone. In this study, we extracted the affected teeth without removing the sclerotic bone when the bone absorption line can be only found between the root and the lesion, and we extracted the teeth as well as the lesion and curetted the granulation tissue when a bone absorption line could be seen between the lesion and the alveolar bone. According to our observation, the position of the bone absorption line can be used as a reference for the selection of sclerotic bone treatment. Sclerotic bone preservation did not increase its volume and density after tooth extraction. Sclerotic bone was composed of highly mineralized tissue with less blood vessels and cells. The position of the bone resorption line can be used as a basis for treatment selection. The high mineralization of the lesion may weaken its anti-infection ability.

## Introduction

Cemento-osseous dysplasia (COD) is a non-neoplastic fibro-osseous lesion of the tooth-bearing regions of the gnathic bones [1]. COD is often incidentally discovered on dental radiography [2]. It is characterized by high-density lesions around the tooth root and in the jaw bone and may also present as mixed transmission/obstruction images. COD is usually asymptomatic in the early stages and may be misdiagnosed as periapical periodontitis and treated with root canal therapy [3,4]. However, such treatment is not effective due to the presence of sclerotic bone around the tooth root [5].­ COD can cause tooth pain, tooth looseness, poor healing after tooth extraction, and osteomyelitis [6]. COD generally leads to tooth loss, osteomyelitis, and osteonecrosis, affecting oral function and facial appearance and affecting whether dental implantation can be carried out due to the presence of sclerotic bone [7]. Therefore, although COD is a non-neoplastic disease, it still causes problems in clinical practice.

## Case presentation

**Figure 1:**
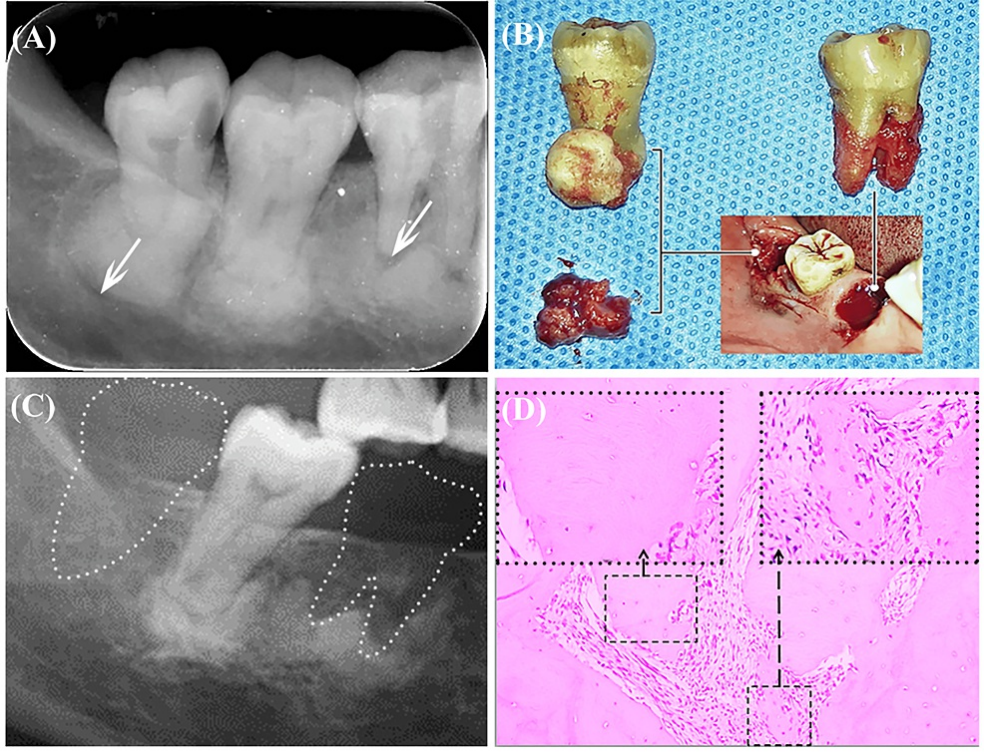
Images from a 65-year-old woman who underwent three treatment methods for cemento-osseous dysplasia of three adjacent teeth (Case 1). (A) Preoperative radiograph. The white arrows indicate transmission-like bone resorption lines. Tooth 46 was extracted, tooth 47 was treated conservatively, and tooth 48 was extracted with sclerotic bone curettage. (B) Extracted teeth and curetted lesion specimens. Tooth 48 has close integration of the root and sclerotic bone, and the curetted tissues from the circular transmission area comprise inflammatory granulation. (C) Radiography taken one year postoperatively showed no lesion recurrence and no change in the shape of the preserved sclerotic bone compared with preoperatively. (D) Hematoxylin and eosin staining of sclerotic bone showed excessive mineralized tissues, few cells and blood vessels, and scattered areas of inflammatory necrosis.

The position of the circular bone resorption line on radiography was used to formulate the treatment plan. If there was no obvious bone resorption line on radiography (Figure 1A, tooth 47), conservative treatment was applied: only need to take a panoramic radiograph once a year to follow the changing of the lesion. If the bone resorption line was adjacent to the root surface and there was no resorption line between the sclerotic bone and the normal jaw bone, only the affected tooth was extracted, while the sclerotic bone was retained (Figure 1A, tooth 46). If the bone resorption line was located between the sclerotic bone and the normal jaw bone, the affected tooth was removed and the sclerotic bone was curetted (Figure 1A, tooth 48). Pathological sections of the sclerotic bone and the bone resorption line were analyzed, and their tissue sources were explored by immunohistochemical staining. The two cases were followed up.

Case 1

A 65-year-old woman presented with recurrent abscess formation and pain in the lower right posterior tooth area. The symptoms had started six months previously, and she had received pulp treatment for tooth 46 in another hospital. She had recently taken antibiotics, but her symptoms had not been substantially relieved. There was no history of treatment for other diseases or administration of special drugs. Clinical examination showed that tooth 46 had an open pulp cavity, the root bifurcation was exposed, and there was a fistula in the buccal gingiva with purulent secretion. Tooth 47 had no obvious abnormalities. Tooth 48 was protruding vertically and had caries on the mesial and adjacent surfaces. Panoramic radiography and cone-beam CT (CBCT) showed oval, high-density shadows surrounding the roots of teeth 46, 47, and 48, suggesting the presence of sclerotic bone. The shadow of circular bone resorption line was located between the tooth root and the sclerotic bone around tooth 46, there was no obvious bone resorption line around tooth 47, and the bone resorption line of tooth 48 was located between the sclerotic bone and the normal jaw bone (Figure 1A). These three adjacent teeth presented three different conditions of COD and thus required three different treatments. Tooth 46 was extracted with the preservation of the sclerotic bone, tooth 47 was treated conservatively, and tooth 48 was extracted with a thorough scraping of the sclerotic bone (Figure 1B). The inflammatory soft tissue (bone resorption line) and sclerotic bone specimens were fixed in formalin, decalcified, paraffin-embedded, and sectioned for histological analysis. One year after surgery, Mimics Innovation Suite 16.0 (Materialise, Leuven, Belgium) was used to calculate the gray value of bone as a representation of bone density. Three-dimensional reconstruction of the lesion shape and calculation of the volume of sclerotic bone were performed.

Hematoxylin and eosin (HE) staining showed inflammatory granulation tissue in the region of the circular bone resorption line, while the sclerotic bone was mainly composed of mineralized bone tissue, with few cells and blood vessels, collagen fibers, immature woven bone (Figure 1D), and scattered areas of inflammatory necrosis. Masson staining showed similar results to HE staining.

**Figure 2:**
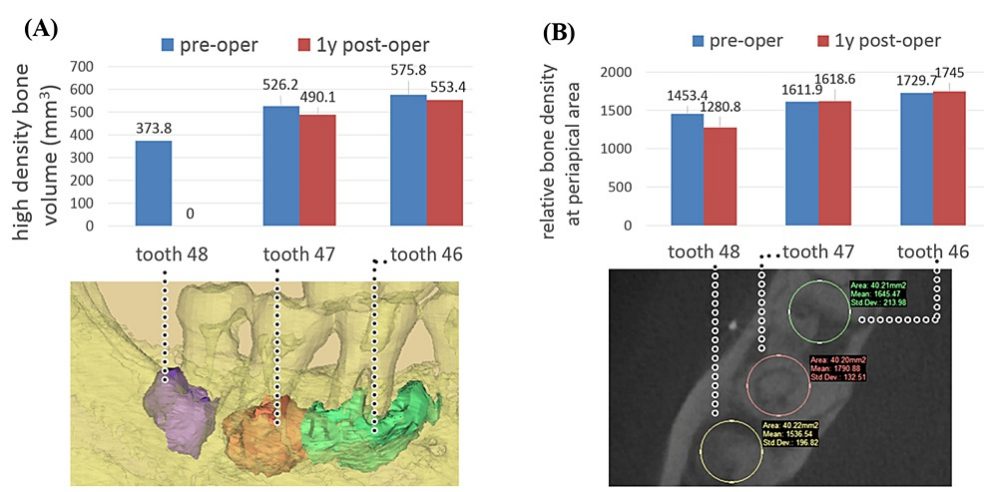
Cone-beam CT examination results from Case 1. (A) Three-dimensional CT reconstruction of sclerotic bone morphology showing that the volume of the lesions did not change markedly in one year. (B) There is no significant difference in the gray value before and after sclerotic bone surgery. The bone density of the region around tooth 48 after sclerotic bone removal is lower than that of the sclerotic bone.

At one year postoperatively, the areas around teeth 46, 47, and 48 had healthy gingiva, and the patient reported no discomfort. Radiography at one year postoperatively showed complete healing of the extraction sockets; the preserved COD sclerotic bone remained asymptomatic, and its overall shape had not changed substantially (Figure 1C). Compared with preoperatively, CBCT showed no marked change at one year postoperatively in the sclerotic bone volume (Figure 2A) and gray value (Figure 2B). The volume of high-density lesions in the apical area of tooth 47 (which was treated conservatively) had not substantially increased. These findings support the "non-neoplastic" description of COD in the 2017 WHO classification. The extraction area of tooth 48 was well healed, and the density of the newly formed bone was not markedly different from that of normal jaw bone. Immunohistochemical results of cementum-related specific proteins, including cementum attachment protein (CAP) and cementum protein 1 (CEMP-1), were positive and the expression of cell proliferation marker Ki67 was negative.

Case 2

**Figure 3:**
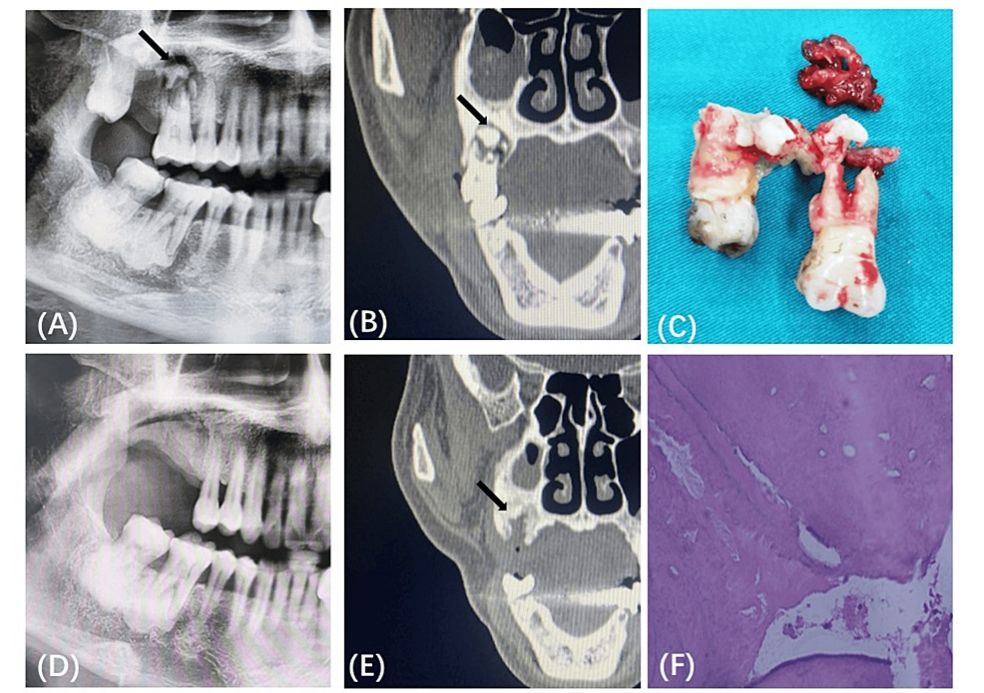
Images from a 64-year-old woman who underwent tooth extraction plus sclerotic bone removal for cemento-osseous dysplasia (Case 2). (A) Preoperative radiography showing sclerotic bone around the roots of teeth 16–18 and transmission-like bone resorption lines. (B) CT images of the sclerotic lesions. The black arrows show the circular transmission area. (C) The extracted teeth and curetted lesions. The white arrow shows the soft tissue curetted from the transmission area. (D) Radiography at five months postoperatively showed good osteogenesis of the maxillary area of teeth 16–18, and stable morphology of the mandibular teeth 47 and 48. (E) CT taken five months postoperatively showed good formation of new bone. (F) Hematoxylin and eosin staining of the sclerotic area showed that the sclerotic bone was composed of highly mineralized bone tissue with inflammatory infiltration.

A 64-year-old woman presented with recurrent abscess formation and pain in the upper right posterior tooth area. The symptoms had started five months previously. Panoramic radiography and CT showed irregular high-density shadows in the apical regions of teeth 16 and 18, which were suspected to be COD (Figures 3A, 3B). An irregular destruction shadow inside the sclerotic bone was speculated to be dead bone, while a circular low-density shadow outside the sclerotic bone was speculated to be inflammatory granulation tissue. In accordance with the position of the bone resorption line, the selected treatment plan was to completely remove the sclerotic bone while extracting the teeth (Figure 3C). Five months after surgery, radiologic and histological analyses were used to evaluate the therapeutic effect.

Re-examination at five months postoperatively showed good wound healing, and the patient reported no discomfort. Panoramic radiography and CT showed good new bone formation in the maxillary surgery area (Figures 3D, 3E). The curetted sclerotic bone and inflammatory tissue were histologically examined. HE staining showed that the sclerotic bone around the roots of teeth 16 and 18 was composed of highly calcified bone tissue with inflammatory infiltration (Figure 3F), while the curetted soft tissue (Figure 3C, white arrow) in the circular transmission area was composed of inflammatory granulation tissue.

COD lesions were also observed around the roots of mandibular teeth 46 and 47 (Figures 3A, 3D); however, as there was no circular bone resorption line on the root surfaces and/or around the sclerotic bone and there was no discomfort, conservative treatment was adopted. The patient was instructed to carefully maintain the health of the teeth and periodontal areas in the affected area. The COD lesions around teeth 46 and 47 have remained stable postoperatively, without osteomyelitis, osteonecrosis, and/or tumor-like growth.

**Figure 4:**
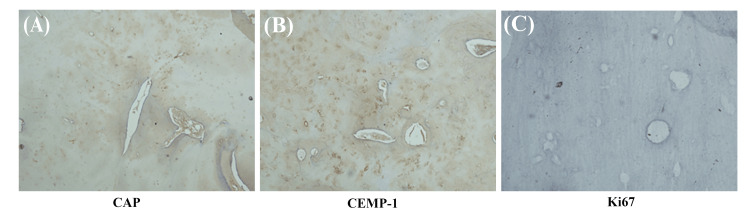
Immunohistochemical staining of the sclerotic lesions in Case 2. The sclerotic lesions in Case 2 are positive for cementum attachment protein (CAP) and cementum protein 1 (CEMP-1), and negative for cell proliferation marker Ki67.

CAP and CEMP-1 were highly expressed in sclerotic bone sections (Figures 4A, 4B). There was no expression of Ki67, which represents the proliferation activity of cells (Figure 4C).

A detailed comparison of the 2005 and 2017 WHO classifications

The description of COD in the World Health Organization (WHO) classification of odontogenic tumors published in 2017 has changed greatly compared with the description published in 2005 [8], mainly regarding the definition and classification of COD. The 2017 edition emphasizes the non-neoplastic nature of COD and does not mention the terms metaplastic bone and idiopathic. Furthermore, the four types of COD described in the 2005 edition were changed to three types in the 2017 edition, and more detailed descriptions of imaging and pathological features have been added. The differences between the 2005 and 2017 editions of the WHO classification of COD are summarized in Table 1.

**Table 1:** Comparison of the 2005 and 2017 editions of the WHO classification of COD. The description of COD in the World Health Organization (WHO) classification of odontogenic tumors published in 2017 [1] has changed greatly compared with the description published in 2005 [8].

Item	2005 edition of the WHO classification	2017 edition of the WHO classification	The differences between the 2005 and 2017 editions
Terminology	Osseous dysplasias (ODs)	Cemento-osseous dysplasia (COD)	The 2005 edition defined them as arising from periodontal tissues but preferred to use the term OD, dropping cemento because cementum and bone are indistinguishable. The 2017 edition reverts to the term COD to recognize them as odontogenic with an origin in the periodontal tissues.
Definition	ODs are idiopathic processes located in the periapical region of the tooth-bearing jaw areas, characterized by a replacement of normal bone by fibrous tissue and metaplastic bone	COD is a non-neoplastic fibro-osseous lesion of the tooth-bearing regions of the gnathic bones	1. The following two items indicate that they can be static lesions: (1) Change "processes" in the 2005 edition to "lesion" in the 2017 edition, indicating that dynamic processes were no longer emphasized and can be stable static. (2) The "metaplastic bone" in the 2005 edition was changed to "fibro-osseous lesion," and the term metaplastic bone was abolished in the 2017 edition, indicating that dynamic changes were no longer emphasized. 2. "Idiopathic" is canceled in the 2017 edition, indicating that it was previously considered to be genetic, but now it is not certain whether it is congenital or acquired, or both are possible. 3. "Periapical" is canceled in the 2017 edition, indicating that it is not limited to the periapical, but can reach the entire periodontal zone and even the jawbone. 4. Increase "non-neoplastic" in the 2017 edition, meaning it will not grow up.
Synonyms	Periapical cemental dysplasia, periapical osseous dysplasia, focal cemento-osseous dysplasia, periapical cementoma	Osseous dysplasia, cemental dysplasia, cementoma	The 2017 edition removes the term "Periapical." Only three simplified synonyms remain.
Epidemiology	OD has a predilection for middle-aged Black females	COD is the most common benign fibro-osseous lesion of the jaws. There is a strong predilection for middle-aged Black women, and an age-adjusted prevalence rate of 5.5% among Black females has been reported	1. Increase "most common" in the 2017 edition, emphasizing that COD has the highest incidence among similar diseases. 2. Increased "strong" in the 2017 edition, indicating that there are large epidemiological differences in age, gender, and ethnicity. 3. Add the term "age-adjusted incidence" in the 2017 edition to avoid the unfair impact of an aging or young society on the incidence of disease.
Classifications	Periapical OD, focal OD, florid OD, familial gigantiform cementoma	Periapical COD, focal COD, florid COD	1. Four types (2005 edition) were changed to three types (2017 edition). 2. Familial gigantiform cementoma is canceled in the 2017 edition.
Clinical features	Gigantiform cementoma can be swollen	Florid type with infection can be swollen and painful	The woven bone in the pathological section is the key point for the identification of COD and ossifying fibroma (OF) and fibrous dysplasia (FD) in the 2005 edition. However, the 2017 edition thinks woven bone is the characteristic that COD is not mature yet, which means it will disappear when COD is mature and it will no longer be used as the distinguishing point of OF and FD. The 2017 edition also mentioned that COD can also be present in the edentulous jaw, while the 2005 edition did not mention it.
Radiation/pathology	A radiolucent halo usually separates the lesions from the surrounding bone and the root surface. OD is considered to originate from the periodontal ligament	A thin radiolucent rim. Macroscopy: The lesions are grossly fragmented, gritty, tan, and brown. Dense hypocellular sclerotic masses may form, especially in florid COD. Osteoblastic rimming.	1. Similarities: (1) Both editions consider that there can be a circular transmission zone between different structures, and the sclerotic bone can be fused with the alveolar bone, but not with the root. (2) Both mentioned that there is no capsule. 2. Differences: (1) The new edition does not mention woven or lamellar bone, nor does it mention histogenesis, indicating that the source of the histogenesis may be controversial. (2) The 2017 edition not only said that the density became higher, but also emphasized the term "mature." The presence of cellular fibrous stroma and osteoids is emphasized; the term "oligocell" is mentioned.
Prognosis and predictive factors	Do not require treatment unless complications occur	Periapical and focal COD: routine dental appointments. Florid COD: close clinical follow-up for complications of osteomyelitis	The 2005 edition considers that none of the four types need treatment unless infection or deformity; the 2017 edition believes that the periapical and focal types require routine follow-up, but the florid type requires close follow-up because the latter is prone to osteomyelitis. However, there is no specific guidance for the treatment of sclerotic lesions after infection and osteomyelitis.

Regarding the treatment of COD, the 2005 WHO classification states that none of the four subtypes of COD need treatment unless there is infection or deformity. In contrast, the 2017 edition states that periapical and focal COD require routine follow-up, while florid COD requires close follow-up to avoid osteomyelitis [9]. However, there is a lack of clear guidance regarding the treatment of highly calcified COD lesions. Thus, it is unclear when the affected teeth should be extracted and whether the diseased sclerotic bone should be removed after the extraction of the affected teeth. The present study evaluated the selection of the treatment method in actual COD cases. The aim of the present study was to provide a reference for the diagnosis and treatment of COD by comparing the differences between the 2005 and 2017 editions of the WHO classification.

## Discussion

The definition and connotations of COD are controversial. COD was previously considered to be an idiopathic lesion characterized by fibrous tissue and metaplastic bone instead of normal bone tissue in the apical region of the jaw bone; however, it is currently considered to be a non-neoplastic fibrous bone lesion in the jaw bone. As the onset of COD is hidden, the condition can easily progress to serious consequences such as tooth loss, osteomyelitis, and even osteonecrosis relatively soon after the occurrence of symptoms.

COD is a disease of abnormal calcification. Compared with normal jaw bone, mature COD lesions have a high degree of bone calcification, which may be related to an inappropriate assessment of the microenvironment and the secretion of minerals by stem cells and osteoblasts. Such excessive mineralization compresses the living space of organic matter, resulting in decreased numbers of cells and blood vessels and decreased immune and repair capabilities [10]. Therefore, regions affected by COD are more prone to osteomyelitis than normal bone [11]. The COD lesions in the present patients caused problems with the teeth or periodontal regions. Therefore, patients with COD need more active protection of teeth to avoid pulpitis and periodontitis and to avoid infection of sclerotic bone. The surgical procedures were selected based on the position of the bone resorption line. Conservative treatment and routine follow-up were performed when there was no obvious bone resorption line. If the bone resorption line was only on the root surface, then the affected tooth was removed, and the sclerotic bone remained in the jaw bone; in fact, it is not easy to curette the sclerotic bone in such cases due to its fusion with normal bone. If the bone resorption line existed in the periphery of the sclerotic bone, then the sclerotic bone and the inflammatory granulation where the bone resorption line was located were curetted at the time of tooth extraction to prevent the development of osteomyelitis.

Prior to 2005, COD was considered a non-neoplastic disease derived from the periodontal ligaments and jaw bones. The 2005 WHO classification stated that this lesion originated from jaw bone tissue, and so the condition was named osseous dysplasia. However, in the 2017 edition of the WHO classification, COD is considered an odontogenic lesion derived from periodontal ligaments, and so the original name was restored (COD). There is controversy regarding the histogenesis of COD. Immunohistochemical examination of sclerotic bone in the present cases showed high expression levels of cementum-related characteristic antigens (CAP and CEMP-1), suggesting that COD is derived from periodontal tissue. In addition, the 2017 WHO classification reduced the number of types of COD from four to three, eliminating the category of gigantiform cementoma; however, it was not explicitly stated whether gigantiform cementoma was integrated into florid COD.

Both the 2005 and 2017 editions of the WHO classification mentioned the presence of a circular transmission zone but denied the fusion between sclerotic bone and the tooth root. However, in Case 1, the COD lesions of tooth 48 were closely integrated with the tooth root. The 2005 WHO classification mentioned that COD lesions contained a large amount of woven, lamellar, and cementoid bone, while the 2017 edition analyzed the pathological manifestations in more detail. It was believed that the woven bones only existed before the COD lesions matured. After maturation, the COD lesions mainly comprised cell fibrous stroma and osteoid and were over-mineralized [12]. The biopsies in the present cases revealed that the sclerotic bone was highly mineralized and mature, with few cells and blood vessels.

The 2005 WHO classification divided the pathological process of COD into three stages. The first phase is the osteolysis and destruction phase, which is shown radiographically as a round low-density transmission area around the periapical region and is easily misdiagnosed as periapical periodontitis. The second stage is the formation of a cementum corpuscle, which is shown radiographically as high-density punctiform or lumpy calcification shadows. The third stage is the calcification maturity stage, with the appearance of large cementum masses and woven tissues seen radiographically as large mass calcification shadows. However, in the 2017 edition of the WHO classification, this dynamic process is no longer mentioned, and dynamic words such as metaplastic bone, replacement, and progress have been eliminated. Instead, COD is described as a lesion, suggesting that the dissolution-osteogenesis process is not an essential feature of the disease, and may even be caused by infection.

The present paper reviewed the differences between the 2005 and 2017 editions of the WHO classification of COD and its connotations. In addition, two case reports were presented to discuss the basis for the selection of treatment methods, which is barely mentioned in the WHO classification. The histopathological characteristics of the COD lesions in these two cases were also analyzed. The present study provides data that may enhance the understanding of COD and emphasize the need for excellent dental hygiene in the affected area to avoid teeth loss and bone defects caused by osteomyelitis.

## Conclusions

The outcome of three treatment methods (conservative, tooth extraction, and tooth extraction with sclerotic bone removal) in accordance with the position of the bone resorption line was evaluated in two patients. There are remarkable differences in the terminology, definition, synonyms, epidemiology, classifications, clinical features, radiation/pathology, prognosis, and predictive factors of COD between the two versions. While there is no clear guidance for the treatment of sclerotic bone. According to our observation, the position of the bone absorption line can be used as a reference for the selection of sclerotic bone treatment. Sclerotic bone preservation did not increase its volume and density after tooth extraction. Sclerotic bone was composed of highly mineralized tissue with less blood vessels and cells. The descriptions of COD in the 2005 and 2017 WHO classifications are quite different. The position of the bone resorption line can be used as a basis for treatment selection. The high mineralization of the lesion may weaken its anti-infection ability. The study provided a treatment strategy for COD based on the position of the bone absorption line by conducting a case analysis and comparing the differences between the 2005 and 2017 editions of the WHO classification.
